# A Functional and Regulatory Network Associated with *PIP* Expression in Human Breast Cancer

**DOI:** 10.1371/journal.pone.0004696

**Published:** 2009-03-05

**Authors:** Marie-Anne Debily, Sandrine El Marhomy, Virginie Boulanger, Eric Eveno, Régine Mariage-Samson, Alessandra Camarca, Charles Auffray, Dominique Piatier-Tonneau, Sandrine Imbeaud

**Affiliations:** 1 Array s/IMAGE, Genexpress, Functional Genomics and Systems Biology for Health, LGN-UMR 7091-CNRS and Pierre & Marie Curie University, Paris VI, Villejuif, France; 2 CEA, DSV, IRCM, LEFG, Laboratory of Genomes Functional Exploration, Evry, France; 3 Université d'Evry Val d'Essonne, Evry, France; 4 Institute of Food Sciences-CNR, Avellino, Italy; 5 Centre de Génétique Moléculaire, UPR 2167, CNRS and Gif/Orsay DNA Microarray Platform (GODMAP), Gif sur Yvette, France; 6 Univ Paris-Sud 11, Orsay, France; Cinvestav, Mexico

## Abstract

**Background:**

The *PIP* (prolactin-inducible protein) gene has been shown to be expressed in breast cancers, with contradictory results concerning its implication. As both the physiological role and the molecular pathways in which *PIP* is involved are poorly understood, we conducted combined gene expression profiling and network analysis studies on selected breast cancer cell lines presenting distinct *PIP* expression levels and hormonal receptor status, to explore the functional and regulatory network of *PIP* co-modulated genes.

**Principal Findings:**

Microarray analysis allowed identification of genes co-modulated with *PIP* independently of modulations resulting from hormonal treatment or cell line heterogeneity. Relevant clusters of genes that can discriminate between [PIP+] and [PIP−] cells were identified. Functional and regulatory network analyses based on a knowledge database revealed a master network of *PIP* co-modulated genes, including many interconnecting oncogenes and tumor suppressor genes, half of which were detected as differentially expressed through high-precision measurements. The network identified appears associated with an inhibition of proliferation coupled with an increase of apoptosis and an enhancement of cell adhesion in breast cancer cell lines, and contains many genes with a STAT5 regulatory motif in their promoters.

**Conclusions:**

Our global exploratory approach identified biological pathways modulated along with *PIP* expression, providing further support for its good prognostic value of disease-free survival in breast cancer. Moreover, our data pointed to the importance of a regulatory subnetwork associated with *PIP* expression in which STAT5 appears as a potential transcriptional regulator.

## Introduction

Breast cancer is one of the most common malignancies in Western countries and is associated with a high mortality rate [1], [2]. Aside from a small subset of patients (∼5%) with inherited genetic alterations, sporadic breast cancer accounts for the majority of all breast cancers and limited knowledge is available about the underlying process of carcinogenesis. It is widely accepted that breast cancer, like most other cancers, develops through the accumulation of genetic aberrations [3]. Some of these changes involve specific genetic loci, determining the activation of oncogenes or the inactivation of tumor-suppressor genes, while others confer genetic instability, which increases the possibility of acquiring additional genetic lesions relevant to tumorigenesis. In the last decades, PIP protein expression has been proposed as a specific and sensitive marker for breast cancer [4]–[7] and further used to support breast origin in metastatic carcinoma of unknown primary origin [8]–[12]. A PIP over-expression was shown in primary and metastatic breast cancers [13], [14], as well as in some breast carcinoma cell lines. However, the exact functions of that protein in mammary tumor progression remain unclear.

In previous work, we reported preliminary conclusions on the PIP properties showing that the protein, a secreted factor known as prolactin-inducible protein (PIP) [13] or as gross cystic disease fluid protein-15 (GCDFP-15)[15], binds to CD4 [16]–[18], exerts a potent inhibition on T lymphocyte apoptosis mediated by CD4/T-cell receptor (TCR) activation [19] and carries a fibronectin-specific aspartyl protease activity [20]. In addition, the *PIP* gene localized on the long arm of chromosome 7 at 7q34 [21] was found to display a variety of rearrangements in numerous solid tumors [22], [23]. Interestingly, we found that the T47D cell line, that constitutively overexpresses *PIP*, exhibits an inverted duplication of the 7q34–q35 region containing the *PIP* gene resulting from a breakage-fusion-bridge (BFB) cycle mechanism initiated within the common fragile site FRA7I [24].

Here, we report an in-depth exploration of the functional and regulatory networks associated with *PIP* gene expression in breast carcinoma cell lines using DNA microarray-based gene expression profiling techniques. Taking advantage of the presence of androgen-responsive elements in the *PIP* gene promoter, breast carcinoma cell lines were analyzed before and after treatment with dihydrotestosterone to modulate *PIP* expression, allowing comparison between the *PIP*-expressing [PIP+] and –non expressing [PIP−] cell profiles. Thus, we identified a series of 205 genes that display significant expression changes between the [PIP+] and [PIP−] subgroups of samples. A representative part of these genes exhibited a good concordance of expression changes when assessed using tailored Q-PCR. A network analysis allowed us to propose that *PIP* gene expression is mainly associated with a decrease of the cell proliferation and migration potential, as well as with an increase of the apoptotic pathway. In addition, the identification of specific STAT5 (Signal Transducer and Activator of Transcription 5) motifs found within promoters of a significant part of the differentially expressed genes suggests that STAT5 could play an important role in the regulatory network associated with *PIP* expression. We also point to other novel modulated pathways that warrant further biological and clinical investigations.

## Results and Discussion

### Characterization of the cellular models

Four breast cancer cell lines presenting different features especially concerning *PIP* expression, hormonal receptor status and invasiveness potential were selected: MDA-MB231, a poorly differentiated and highly invasive cell line, MCF7, T47D and VHB1, which are known to be differentiated and non-invasive breast cancer cell lines [25].

As *PIP* expression was shown to be increased by androgens [26], we first analyzed the expression of the androgen receptor (*AR*). Moreover, as the estrogen receptor (*ER*) expression is currently used as a potential marker to classify breast cancer samples, and the ER-positive tumors are often found associated with a better outcome than the ER-negative ones [27], [28], we also analyzed the status of this hormonal receptor in the four cell lines. RT-PCR analysis of *AR* expression in the four cell lines indicated that MDA-MB231 is AR-negative and MCF7 AR-positive (data not shown). As expected both T47D and VHB1 cells were AR-positive. Similarly, the expression of *ER* was found positive in MCF7, T47D and VHB1 and negative in MDA-MB231. The phenotype of the cell lines further used for DNA microarray-based gene expression profile studies is summarized in Table 1.

**Table 1:** PIP and hormonal receptor status in breast cancer cell lines.

	*PIP without DHT*	*PIP with DHT*	*AR*	*ER*
**T47D**	++	++	+	+
**VHB1**	−	+	+	+
**MCF7**	−	−	+	+
**MDA-MB231**	−	−	−	−

Relative expression of *PIP*, androgen receptor (*AR*) and estrogen receptor (*ER*) assessed by RT-PCR in each cell line. The *PIP* expression was determined with or without DHT treatment. The cell lines were classified in 3 distinct categories, corresponding to no (−) expression or a basal (+) or a high (++) level of expression for each gene, respectively.

**Figure 1:**
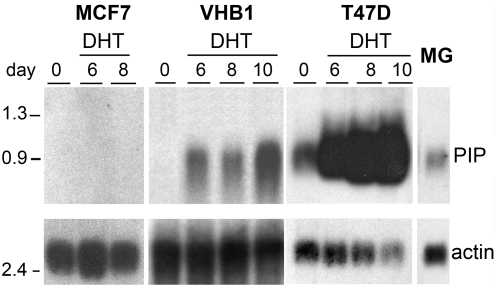
*PIP* expression analysis after DHT treatment in breast carcinoma cell lines and normal mammary gland. Relative abundance of PIP mRNA was assessed by Northern Blot analysis (upper panels). Total RNAs were extracted from normal mammary gland (MG, 15 µg) and from three breast carcinoma cell lines (MCF7, T47D and VHB1, 50 µg), at several days (0, 6, 8 and 10 days) after DHT treatment. The relative β-actin expression levels in each sample are shown (lower panels).

Androgen treatments were used to induce *PIP* expression. Breast cancer cell lines were grown in presence or absence of 10 nM of dihydrotestosterone (DHT) for various periods, and *PIP* gene expression was analyzed by northern blot (Figure 1). No detectable *PIP* expression was found in MCF7 despite the expression of *AR*; similar results were obtained in MDA-MB231 even after 10 days of androgen treatment (data not shown). In contrast, in T47D which constitutively expresses *PIP*, DHT treatment for 6, 8 and 10 days increased *PIP* expression about 2.5, 4 and 4.9 times, respectively, whereas in VHB1 that did not exhibit detectable endogenous expression, DHT treatment induced a consistent *PIP* expression. A low *PIP* expression level was observed in mammary gland samples (MG) taken from healthy women (Figure 1). Identical phenotypes were observed by RT-PCR (data not shown). In the present study, our strategy was to take advantage of the differences in hormonal receptor expression level and invasiveness potential of the distinct cell lines in order to predominantly focus on the gene expression modulations associated with *PIP* expression, but independently of the possible influence of particular genetic backgrounds. Thus, the samples were categorized in either a [PIP+] or a [PIP−] subgroup and used for subsequent analyses.

### Experimental design and statistical power simulations

Gene expression profiles were collected in duplicate from a total of 32 RNA samples derived from 4 independent cultures and RNA preparations of the 4 breast carcinoma cell lines cultured without (J0) and with DHT for 7 days (J7).

The a priori statistical power of the gene expression dataset was measured as the probability of obtaining statistical significance when true biological differences exist between the compared groups of samples (1 - β; true positive rate). A conventional power analysis requires the designation of parameters such as the anticipated variability of individual measurements for all genes within each biological group (σ), the total sample size (n, n1 & n2), the magnitude of the effect to be detected (Φ) and the acceptable false positive rate (significance level α). It allows verifying which subgroups of samples are likely to provide the most comprehensive relevant information and that enough samples are compared to meet the objectives of the study.

Thus, samples were divided in 2 subgroups according to their *PIP* expression level (Table 1). MDA-MB231 (J0 and J7), MCF7 (J0 and J7) and VHB1 (J0) samples were considered as [PIP−] and all others as [PIP+] samples. Some additional analyses were conducted using 3 phenotype classes [PIP−], [PIP+] and [PIP++] instead of 2. In this case, the [PIP++] subgroup contained T47D (J0 and J7) samples as the *PIP* expression level is significantly higher than in VHB1 (J7) and can influence differently the gene expression profiles (Figure 1).

Statistical power (1-β) was computed for a two-class comparison, detecting a true 1.5-fold mean difference between either the [PIP+] or [PIP++] group of samples and the [PIP−] group of samples at a significance level (α) of 0.01, considering a total sample size of 64 or 56 respectively with n2/n1 = 0.6 or 0.4 (Table 2), and an expected variability within each biological sample group σ_median_< = 0.30. The statistical power was estimated to be satisfactory, with a limited proportion of false negatives (β = 0.01 to 0.03, Table 2), while consistent with a small number of spurious discoveries (cf. Table 3).

**Table 2:** Statistical power simulations of the gene expression dataset.

[PIP++] vs [PIP−] n = 16 vs n = 40	[PIP+] vs [PIP−] n = 24 vs n = 40	Ratio
0.29	0.41	**1.2**
0.78	0.90	**1.35**
**0.97**	**0.99**	**1.5**
**1**	**1**	**1.8**

The statistical power (Z; 1- ß) is the probability of obtaining statistical significance in comparing gene expression. Simulations for unpaired two-class comparison statistics are described in the Materials & Methods. Calculation includes the following parameters:

- a significance level (α) of 0.01.

- the observed biological variability (σ).

- a sample size (n) from individual [PIP++], [PIP+] and [PIP−] samples.

- a true difference (i.e. 1.2, 1.35, 1.5 or 1.8) in mean expression ratios between the respective classes.

**Table 3:** False discovery rate of the gene expression dataset.

[PIP++] n = 16	[PIP+] n = 24	[PIP−] n = 40	Ratio
3.95e−02	1.57e−02	2.74e−03	**1.2**
1.92e−03	1.99e−04	2.42e−06	**1.35**
8.58e−07	8.58e−07	3.28e−10	**1.5**

Expected False discovery rates (FDR) that may be anticipated from a gene expression comparisons from the [PIP++], [PIP+] and [PIP−] subgroups of samples.

The simulations thus suggested that only a negligible proportion of the information relevant to the question addressed would be missed in the class comparisons, and provided a high confidence toward the differentially expressed genes identified.

### Measure of the range of biological variability in samples

**Figure 2:**
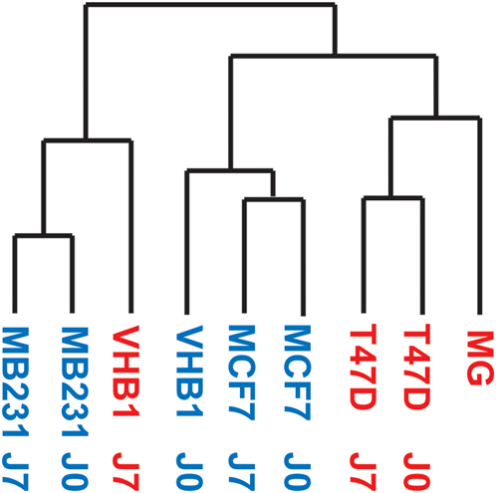
Range of biological variability of the gene expression dataset. Similarity dendrograms (Pearson correlation) resulting from unsupervised hierarchical clustering of DHT-treated (J7) or -untreated (J0) breast carcinoma cells based on the global gene expression matrix. [PIP+] and [PIP−] cells are indicated in red and blue, respectively.

As the cell line intrinsic properties may obscure expression patterns related to *PIP* gene expression, we appreciated the range of biological variability through unsupervised clustering of the entire gene expression profiles (Figure 2). Similarity measures between genes were computed using a Pearson correlation. Clusters were defined by an average linkage clustering method. No clear cluster was observed according to either the *PIP* gene expression level or the ER status, the resulting dendrograms of the samples probably reflecting predictable biological variability between cell lines. As shown in Figure 2, samples were clustered in two distinct groups, one containing DHT-untreated (J0) or -treated (J7) MDA-MB231 and VHB1 J7 samples, the other MCF7 (J0 and J7), VHB1 J0, T47D (J0 and J7) and normal mammary gland samples. Except for the VHB1 samples, serially treated cell line samples tended mainly to cluster together, independently from DHT treatment or *PIP* expression. This indicates that the genome-wide expression profile changes induced by the hormonal treatment may be less prominent than the inherent observed cell line differences. The transcriptome data analysis strategy was therefore designed to assess the likelihood of detecting reliably significant gene expression differences linked to variations in *PIP* expression.

### Identification of the genes co-modulated with *PIP* expression

Beforehand, several statistical differential comparisons were performed to first highlight gene expression modulation that may be unrelated to the *PIP* gene influence, but potentially resulting from the cell line heterogeneity itself. The analysis was done comparing the expression profiles of the 3 cell lines associated with a [PIP−] phenotype (i.e. MCF7, VHB1 and MDA-MB231) without DHT treatment in order to identify specific unique gene expression. This analysis pointed out 85% of the genes (7,996 clones) that were found not significantly differentially expressed between the 3 cell lines (p = 0.01). The corresponding gene list was used as a reference for subsequent statistical analyses with the drawback that a fraction of them will escape detection of differential expression in relation with *PIP* in subsequent analyses, being confounded by differences in the genetic background of the individual cell lines, but ensuring that the gene modulations identified are strictly related to *PIP* expression. Two- and three-class comparisons of mean relative expression levels were then performed gene-by-gene between [PIP++], [PIP+] and [PIP−] subgroups using a combination of *t*- and *F*- statistic approaches and yielded complementary lists (See Table S1), including a list of 606 clones (L606, two-class, data not shown) and a list of 235 clones (L235, three-class, see Table S2). The 3-class comparison was privileged to take into account the additional modulations of expression that could occur between samples with a moderate [PIP+] or a high [PIP++] expression and to focus our exploration on genes that may be found co-regulated in relation to the *PIP* expression level changes. The split of *PIP*-expressing samples in two separate subgroups of samples raised the strength of the statistical analysis and led to the identification of a more extensive and explicit list of modulated genes, as 44% of the clones in L235 were not detected in two-class comparison and therefore not included in L606. In addition, these genes are unlikely to correspond to genes modulated by DHT treatment independently from *PIP* expression, since both [PIP+] and [PIP++] subgroups of samples consist of treated samples and the [PIP++] subgroup contains a balanced number of treated and untreated samples.

The genes represented in L235 were annotated using the Unigene Cluster Ids identifiers [29]. L235 corresponds to 205 unique genes (193 unique named genes) (L235; see Table S2), including 92 up-regulated named genes (64%) and 51 down-regulated named genes in the [PIP+] group when compared with the [PIP−] group, with a fold-change over 1.35. More than one third of the selected genes were associated with a fold-change ranging from 1.35 to 1.5; thus, the effective statistical power was computed to evaluate the reliability of detection of such slight gene modulations for both two- and three-class comparisons. We found the computed power to be satisfactory, being over 90% and 78%, thereby ensuring the reliable detection of these small variations in gene expression between [PIP+] and [PIP−] samples and between [PIP++] and [PIP−] samples, respectively (Table 1 & Table 2). The false positive rate associated with this threshold ratio was estimated to be lower than 0.2% in all subgroup comparisons (Table 3), confirming that these slightly modulated genes could be taken into account confidently.

**Figure 3:**
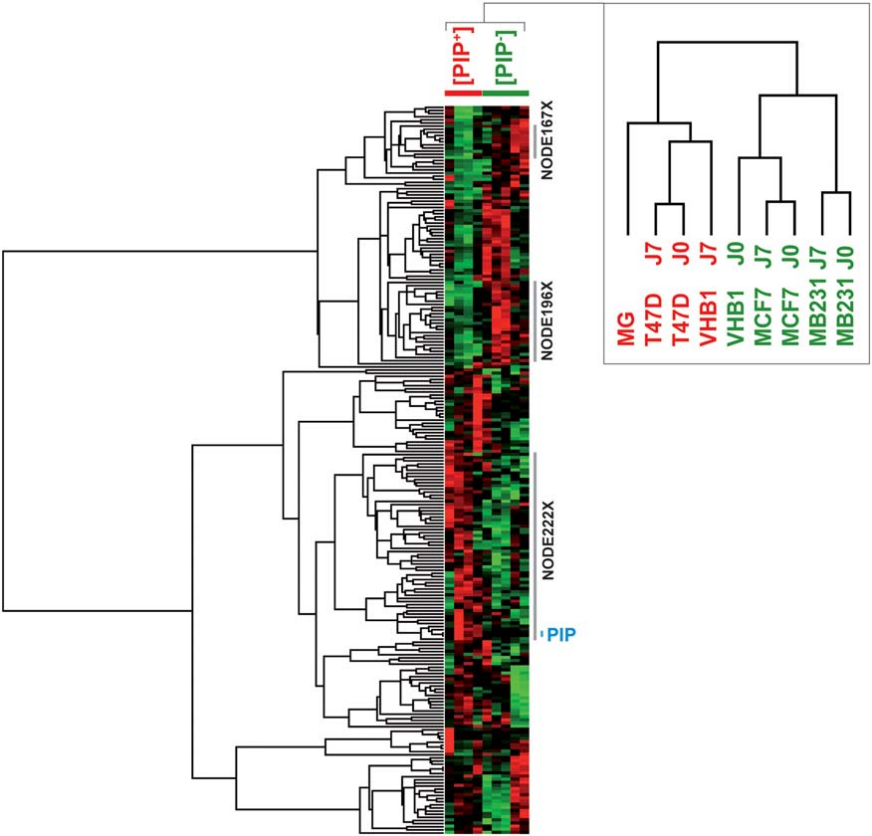
Hierarchical clustering of the differentially expressed genes. Unsupervised hierarchical clustering of all samples for the genes found significantly differentially expressed (L235) between [PIP+] and [PIP−] phenotypes, and modulated in relation with the *PIP* expression. Genes (row) and samples (columns) are clustered independently using uncentered Pearson correlation metrics. [PIP+] and [PIP−] cell lines are indicated in red and green, respectively. The top-ranked relevant gene clusters (NODE 167X, NODE 196X and NODE 222X) selected using *t*-statistics with permutation-based adjustment (n = 10,000; α = 0.05) are indicated by color bars. The presence of the *PIP* gene is pointed.

To further probe the ability of different subsets of the genes represented in L235 to discriminate between [PIP+] and [PIP−] phenotypes, supervised hierarchical clustering of the expression profiles was performed (Figure 3). Gene clusters with related expression patterns were clearly discernable, consistently pointing out differences between the PIP–expressing and non-expressing samples. Precisely, the samples are divided in two main groups according to their *PIP* expression phenotype. This observation contrasts with the sample similarity dendrogram previously obtained using the whole gene expression matrix (Figure 2). Clusters of gene modules that appeared the most relevant to differentiate [PIP+] and [PIP−] subgroups were identified using *t*-statistics with a permutation-based adjustment of the gene expression matrix (n = 10,000 and α = 0.05). The top-ranked clusters were NODE222X of 60 clones (50 named genes and 7 not assigned to any Unigene cluster Id) found up-regulated in the [PIP+] subgroup (*t*-stat = −4.57; p = 2.5×10^−4^) and NODE196X and NODE167X containing 26 and 11 clones (23 and 8 named genes, respectively, and 3 not assigned to any Unigene cluster Id in each node), which conversely represent clusters of genes up-regulated in the [PIP−] subgroup (*t*-stat = 4.16 and 2.89 and ; p = 8.7×10^−4^ and 2×10^−2^) (Figure 3).

### Validation of the microarray gene expression data

The accuracy and reliability of the results obtained with microarrays was tested by quantitative RT-PCR (Q-PCR) using a tailored TaqMan Low Density Array (LDA). The relative gene expression levels (RQ = 2^−ΔΔCt^, [30]) in [PIP+] and [PIP−] samples were normalized to that of the peptidylprolyl isomerase A (*PPIA*) housekeeping gene and relative to the median value of all samples taken as calibrator reference. The data are expressed as the RQ ratios in [PIP+] versus [PIP−] samples.

Thirty-two genes (28 from list L235, 4 from lists L578 and L2231, see Table S1) were chosen for validation of microarray data. Nine additional genes were selected among those found not significantly differentially expressed (Table 4). Comparison of microarray and Q-PCR results after z-statistics with FDR adjustments indicated a good agreement: 28 of the 32 (87%) differentially expressed genes detected with microarrays were fully validated by Q-PCR (Table 5). The remaining differentially expressed genes were considered as false positive results: two of them were associated with an inverted Q-PCR expression ratio compared to that obtained with microarrays and the 2 others were not found significantly differentially expressed when analyzed by Q-PCR. Finally, four of the nine genes not detected as differentially expressed with microarrays and found discordant when analyzed by Q-PCR are likely to represent microarray false negative results or false positive results of one or the other technology (Table 5).

**Table 4:** Descriptive list of the genes selected for biological validation by quantitative RT-PCR (Q-PCR).

N°	Symbol	Clone ID	Intensity [PIP+]	Intensity [PIP−]	Ratio PIP+/PIP	Min *p*-value	Max *p* -value
**1**	**PIP**	4295801	23801	1753	13.83	0.00E+00	0.00E+00
**2**	**CDKN2A**	2988668	2549	526	2.64	6.79E−08	0.00E+00
**3**	**CD82**	2959683	3164	1178	1.62	8.55E−09	0.00E+00
**4**	**DSCR1**	3944959	4559	2731	1.92	2.66E−08	0.00E+00
**5**	**RERG**	3357341	4483	1466	5.35	8.01E−11	0.00E+00
**6**	**ACAT1**	4278329	4558	1844	1.89	1.05E−04	0.00E+00
**7**	**HRASLS3**	3051149	9275	3243	1.88	7.46E−06	0.00E+00
**8**	**BDH1**	2822178	3874	1526	1.55	1.94E−04	2.54E−13
**9**	**MPHOSPH6**	3997566	6085	4122	1.67	8.52E−07	1.24E−13
**10**	**PEA15**	3346270	2949	2013	1.60	2.88E−06	7.32E−10
**11**	**RFC4**	3537752	6569	3081	1.45	6.57E−07	2.22E−16
**12**	**TFRC**	587896	4484	2724	1.48	2.35E−07	6.55E−12
**13**	**NDUFB5**	3997377	9508	6340	1.51	2.94E−07	0.00E+00
**14**	**NDUFS2**	3138814	6354	2818	1.60	1.44E−08	6.66E−16
**15**	**MRPL45**	3951804	3712	2989	1.36	3.45E−04	4.85E−08
**16**	**BAD**	345703	2652	1954	1.34	8.91E−04	1.67E−08
**17**	**NFRKB**	131626	1489	1065	1.49	6.04E−08	0.00E+00
**18**	**BCL2** *	232714	955	1989	−2.08	1.26E−02	5.23E−08
**19**	**TGFBI**	2958878	659	1316	−1.96	2.99E−06	0.00E+00
**20**	**NRAS**	3826638	2308	3126	−1.72	8.07E−06	2.74E−10
**21**	**CTSL**	4295635	2847	4049	−1.64	6.25E−10	2.22E−16
**22**	**CD44**	3638681	4176	5002	−1.52	1.66E−06	2.71E−13
**23**	**COL6A2** *	3347413	882	2151	−2.43	6.30E−05	0.00E+00
**24**	**CDKN1A** *	2821049	1429	1177	−1.22	7.04E−01	1.53E−01
**25**	**MYC** *	417226	3419	7352	−2.17	1.90E−03	1.61E−03
**26**	**NBN**	3997534	1283	1843	−1.32	1.54E−03	2.28E−04
**27**	**CTPS**	3507350	3905	4637	−1.54	4.83E−08	1.09E−14
**28**	**IDH3A**	2989636	2026	3468	−1.47	8.65E−07	1.69E−13
**29**	**BAG1**	2823774	1195	1022	−1.41	1.38E−03	8.09E−06
**30**	**EEF1B2**	3353094	7955	15656	−1.82	1.06E−10	0.00E+00
**31**	**IQGAP1**	770999	1544	2469	−1.37	1.97E−03	2.76E−07
**32**	**ITGB6**	759142	852	444	2.17	4.54E−11	0.00E+00
*33*	*CCNE1*	*3637746*	5487	6216	n.d	not significant	
*34*	*RB1*	*668108*	2685	2349	1.01	not significant	
*35*	*SERPINF1*	*2961120*	867	862	n.d	not significant	
*36*	*COL6A1*	*3506644*	786	820	−1.25	not significant	
*37*	*MAD2L1*	*2964388*	4329	5068	−1.25	not significant	
*38*	*LAMA3*	*298718*	1814	1705	1.06	not significant	
*39*	*EGFR*	*151475*	903	761	1.19	not significant	
*40*	*TGFB1*	*3356605*	3396	3463	−1.02	not significant	
*41*	*TP53*	*3544714*	3027	3351	−1.18	not significant	

Thirty-two genes found differentially expressed (displayed in bold) and 9 additional genes with no significant differential expression (displayed in italics) using microarrays (α = 0.01) were selected for further biological validation. For each gene symbol, clone ID and median intensity values (displayed in arbitrary unit) of [PIP+] and [PIP−] cell lines are indicated. The relative expression levels recorded with microarrays are displayed as the ratio between [PIP+] and [PIP−] samples, and the values specified as negative (down-regulated) or positive (up-regulated). Adjusted *p* values were computed using z statistics with false discovery rate corrections (α = 0.05). Min *p* value and Max *p* value refer to lower and upper bound *p* values, respectively.

* Differentially expressed genes from lists L578 (*BCL2*, *MYC*) (Table S1) and L2231 (*BCL2*, *CDKN1A*, *COL6A2*, *MYC*) (See Table S1).

**Table 5:** Q-PCR validation of the expression of the 41 selected genes in cell lines.

	Symbol	Assay ID	Ct [PIP+]	Ct [PIP−]	Ratio PIP+/PIP−	*p*-value
**Fully validated genes**	**PIP**	Hs00160082_m1	18.09	34.94	33905	0.00E+00
	**CDKN2A**	Hs00233365_m1	23.79	40.00	1862	8.22E−15
	**CD82**	Hs00174463_m1	22.52	29.71	101.36	0.00E+00
	**DSCR1**	Hs00231766_m1	23.83	26.70	5.68	0.00E+00
	**RERG**	Hs00262869_m1	24.30	26.29	4.37	5.02E−11
	**ACAT1**	Hs00608002_m1	22.34	25.03	4.01	0.00E+00
	**HRASLS3**	Hs00272992_m1	21.76	23.97	3.43	6.44E−15
	**BDH1**	Hs00366292_m1	25.02	26.52	2.81	3.36E−11
	**MPHOSPH6**	Hs00757922_g1	22.86	24.59	2.66	3.37E−08
	**PEA15**	Hs00269428_m1	22.16	23.84	2.58	6.60E−10
	**RFC4**	Hs00427469_m1	23.42	24.82	2.46	2.52E−09
	**TFRC**	Hs99999911_m1	20.03	22.03	2,40	6.41E−09
	**NDUFB5**	Hs00159582_m1	20.99	22.22	2.31	3.53E−08
	**NDUFS2**	Hs00190020_m1	22.24	23.76	2.28	4.54E−08
	**MRPL45**	Hs00260597_m1	24.32	25.71	2.18	7.73E−07
	**BAD**	Hs00188930_m1	24.16	25.21	2.05	4.17E−06
	**NFRKB**	Hs00196269_m1	25.02	25.94	1.96	8.75E−06
	BCL2*	Hs00608023_m1	35.06	26.81	−1437	0.00E+00
	**TGFBI**	Hs00165908_m1	29.53	26.42	−12.50	2.22E−16
	**NRAS**	Hs00180035_m1	23.07	20.77	−3.31	1.69E−07
	**CTSL**	Hs00377632_m1	25.65	23.37	−3.00	6.25E−05
	**CD44**	Hs00174139_m1	26.34	25.07	−2.90	5.34E−05
	COL6A2*	Hs00365167_m1	28.91	27.23	−2.79	3.22E−05
	CDKN1A*	Hs00355782_m1	24.20	22.63	−2.01	1.21E−02
	MYC*	Hs00153408_m1	25.21	23.32	−1.99	9.55E−04
	**NBN**	Hs00159537_m1	24.02	23.33	−1.73	9.02E−03
	**CTPS**	Hs00157163_m1	25.03	23.91	−1.60	2.06E−02
	**IDH3A**	Hs00194253_m1	23.53	23.39	−1.55	2.65E−02
	*CCNE1*	*Hs00233356_m1*	26.19	26.36	1.11	5.26E−01
	*RB1*	*Hs00153108_m1*	24.94	25.24	1.11	5.58E−01
	*SERPINF1*	*Hs00171467_m1*	29.96	29.45	−1.23	2.53E−01
	*COL6A1*	*Hs00242448_m1*	27.83	27.44	−1.20	3.14E−01
	*MAD2L1*	*Hs00829154_g1*	25.60	25.61	−1.01	9.65E−01
**False discoveries**	**BAG1**	Hs00185390_m1	23.82	23.75	−1.04	8.21E−01
	**EEF1B2**	Hs00253438_m1	25.34	24.78	−1.33	1.28E−01
	**IQGAP1**	Hs00182622_m1	23.13	23.02	−1,10	5.79E−01
	**ITGB6**	Hs00168458_m1	29.91	27.25	−5.75	1.00E−02
	*LAMA3*	*Hs00165042_m1*	26.54	28.47	3.08	5.05E−12
	*EGFR*	*Hs00193306_m1*	28.12	29.40	2.50	4.22E−08
	*TGFB1*	*Hs00171257_m1*	27.32	23.70	−5.83	9.18E−13
	*TP53*	*Hs00153340_m1*	23.22	21.83	−2.23	1.89E−04

Q-PCR analysis was done according to the Material&Methods on the same samples set used in microarray analysis. Gene symbol and TaqMan assay (assay ID) are indicated. The genes that were found differentially expressed (L235) or not significantly modulated using microarray are displayed in bold and italics, respectively, and classified as fully validated genes or potential false discoveries considering the Q-PCR results. The Ct values correspond to the median of threshold cycles of [PIP+] and [PIP−] samples. The expression changes (ratio) between the [PIP+] and the [PIP−] samples are specified as negative (down-regulated) or positive (up-regulated) values. Adjusted *p*-values were computed using z statistics with false discovery rate corrections (α = 0.05).

Taken together, these results show that most of the genes identified by microarrays were validated and only few genes were found to be false positive results. Thus, expression of the genes in L235 derived from the three-class comparison correlates directly or inversely with *PIP* modulations. It provides a faithful representation of the breast cancer cellular model and therefore a solid basis for further functional exploration of the results.

### Functional annotation of the differentially expressed genes

Functional analysis was performed using the Ingenuity Pathway Analysis (IPA, version 4.0) tool which relies on a knowledge database of curated functional and regulatory interactions extracted from the literature and provides integrated graphical representation of the biological relationships between genes and gene products. Two distinct analyses were performed based on Locuslink ID gene identifiers, considering separately up- and down-regulated genes from list L235 (Table 6). The *p*-values relative to the most enriched functions appeared highly significant (α = 0.05, Fisher's exact test). A total of 48 and 43 significantly over-represented biological functions were identified in the up- and down-regulated gene lists, respectively. Among them, 37 are overlapping. The significance was higher for functions associated with down-regulated genes. The 20 most relevant functions identified with down-regulated genes and the corresponding *p*-values for both analyses are reported in Table 6.

**Table 6:** Functional analysis of *PIP* co-modulated genes.

Molecular function	Up-regulated genes	*p*-value	Gene, *n*	Down-regulated genes	*p*-value	Gene, *n*
Cell cycle		**5.35E−12 to 8.79E−5**	34		**2.76E−17 to 2.11E−7**	39
Cancer		**1.23E−11 to 9.72E−5**	68		**8.72E−15 to 3.92E−7**	51
Hematological System Development And Function		**1.56E−11 to 9.04E−5**	43		**2.67E−10 to 3.92E−7**	29
Cell Growth and Proliferation		**8.52E−11 to 9.54E−5**	67		**1.15E−15 to 4.14E−7**	55
Cell Death		**1.33E−10 to 9.72E−5**	62		**1.96E−12 to 3.58E−7**	47
Tissue Morphology		**2.33E−10 to 6.97E−5**	38		**6.63E−11 to 2.27E−7**	33
Gene expression		**2.75E−10 to 3.45E−5**	53		**2.55E−14 to 3.09E−7**	40
Cell Morphology		**4.28E−10 to 8.66E−5**	38		**1.50E−14 to 2.67E−7**	40
Cellular Development		2.69E−9 to 9.54E−5	49		**1.41E−14 to 3.92E−7**	47
Immune and Lymphatic System Development and Function		9.16E−9 to 9.04E−5	41		**2.67E−10 to 3.92E−7**	24
Immune Response		1.81E−8 to 2.96E−5	39		**2.67E−10 to 5.74E−9**	20
Cellular Movement		5.37E−8 to 7.51E−5	37		**8.04E−13 to 3.92E−7**	39
Connective Tissue		1.85E−7 to 1.76E−5	26		**5.45E−12 to 1.50E−7**	25
Cell to Cell Signalling and Interaction		1.91E−7 to 7.17E−5	30		**5.30E−10 to 2.48E−7**	36
Reproductive System Disease		8.02E−7 to 3.39E−5	19		**1.47E−11 to 3.92E−7**	24
Tumor Morphology		1.10E−6 to 5.85E−5	15		**1.20E−10 to 7.09E−8**	23
DNA Replication, Recombination and Repair		1.47E−6 to 8.79E−5	17		**9.22E−14 to 2.66E−8**	28
Cell Signalling		2.34E−5 to 2.34E−5	5		2.16E−9 to 2.16E−9	14
Cellular Assembly and Organization		*>7.51E−5*			**2.16E−11 to 2.97E−7**	22
Tissue Development		*>7.51E−5*			**5.30E−10 to 4.14E−7**	36

List of the statistically relevant top twenty of over-represented biological functions. Distinct analyses were performed using the IPA tool (version 4.0) for up- and down-regulated genes. For each function, the computed *p*-values are reported as well as the total number of genes. A *p*-value for a given process is computed using a one-side right-tailed Fisher exact test (α = 0.05) by considering the total number of up- or down-regulated genes from L235 and the number of genes that are known to be associated with that process in the IPA knowledge base. As a function may be divided in several sub-functions (for instance, the functions interphase, S phase and G1 phase and G0/G1 phase refer to the unique cell cycle function), the statistical results are displayed as an interval of *p*-values reflecting the range computed for each sub-function. The biological processes are ranked according to the *p*-value of up-regulated genes. The *p*-value appear in bold if the upper limit of the interval is lower than 1e−10.

Thus, cancer, cell cycle, cellular growth and proliferation related functions appeared to be in the top five of highest-level functions highlighted in both analyses according to the assigned *p*-value. Cell death was also associated with significant expression changes in genes co-modulated with *PIP* (1.33e−10 to 9.72e−5 for up-regulated genes and 1.96e−12 to 3.58e−7 for down-regulated genes). Taken together, these results suggest that tumor proliferation might be deeply impacted by modulations of gene expression levels between the [PIP+] and the [PIP−] samples. Moreover, significantly enriched gene classes related to down-regulated genes in [PIP+] versus [PIP−] samples are highly indicative of processes involving cell morphology and movement. The prominent functions associated with theses classes are cell morphology, tissue morphology, tumor morphology, cellular movement, cell to cell signalling and interaction, and cellular assembly and organization (Table 6). These biological functions are also found significantly over-represented in the up-regulated gene list even though the associated *p*-values are slightly smaller but statistically relevant (*p*-value lower limit≤1e−6). A large number of genes involved in cellular movement partly overlapped with cancer-related genes. Altogether these gene expression modulations might influence cell death or tumor invasiveness.

### Identification of molecular networks associated with *PIP* gene expression modulations

We next investigated biological relationships between genes and gene products by performing a network analysis for the genes represented in L235. A total of 126 unique genes in L235, called focus genes, were mapped to genetic networks as defined by the IPA tool. Nine networks were found significantly enriched with scores ranging from 9 to 19 (data not shown), the probability for a network to be selected by chance (score<3) decreasing when its corresponding score value increases. As network identification using the IPA tool may be strongly dependent upon size and content of the input gene list used, further analyses were conducted using independent gene lists for the up- and down-regulated genes in L235. This analysis led to the identification of ten and eleven networks for up- and down-regulated genes, respectively (data not shown). These networks were associated with the same biological functions (i.e. cancer, cell death, cell cycle, cellular growth, gene expression, proliferation and tissue morphology) exhibiting higher scores (15–23 and 8–23 for the top 6 up- and 4 down-regulated gene networks, respectively) as those previously identified using the whole gene list L235 (Table 7).

**Table 7:** Global network analysis of differentially expressed genes.

	id	Genes	Score	Focus genes	Top functions
**Up-regulated gene analysis**	1	ACHE, BCL2L1, BCL2L11, CCL17, **CCRN4L**, CD14, **CD82**, **COMMD9**, CP, **DHRS3**, **DSCR1**, **DUSP14**, **EFNA1**, FOXO1A, GAS6, HSD11B1, **LGALS8**, LTBR, **LTF**, MAP3K7IP2, NFKB1, **NFRKB**, NOTCH4, **NR1H3**, PDGFA, PPP4C, **PSEN2**, PTX3, SOD2, TAP1, TCF3, TNF, **TNNT1**, **UCP3**, **VDAC3**	23	**15**	Tissue Morphology, Cell Death
	2	**ATP6AP2**, **BAD**, **BDH1**, BMYO, **CCND3**, **EIF4A1**, **EIF4A2**, EIF4B, EIF4E, EIF4G1, EIF4G2, EIF4G3, FGB, FGF2, FGG, GH1, **GLUD1**, **GLUL**, GOLGA2, GORASP2, HMGA2, **ICAM1**, IGFBP2, ITM2B, KRAS, LAMA5, MCAM, MKNK1, **MMP14**, PDCD4, PRDX4, REN, S100A4, **TEAD4**, **TMED2**	17	**12**	Protein Synthesis, RNA Post-Transcriptional Modification, Gene Expression
	3	**ACAT1**, **ACVRL1**, **ALG3**, CAP1, **CAP2**, **EPB41L1**, **FDFT1**, FN1, GCNT1, IGFBP7, **ITGB6**, ITGB8, LEP, **LTBP1**, ND1, ND2, ND4, ND6, ND4L, NDUFS1, **NDUFS2**, NDUFS3, NDUFS4, NDUFS5, NDUFS6, NDUFS8, NDUFV2, PTEN, RB1, **RPN2**, RRM1, **SC5DL**, SPI1, **SRPR**, TGFB1	17	**12**	Energy Production, Molecular Transport, Genetic Disorder
	4	AMD1, CBX5, **CCND3**, **CDKN2A**, CITED2, **CLDN6**, DCTN4, DHFR, DMTF1, E2F6, EED, EPC1, ESRRA, EZH2, **GOT1**, HIST3, HMGB2, KLF4, **MTCH2**, NFE2L2, PCGF4, **PDHX**, PMF1, **PRAME**, RAD51AP1, RECQL4, **REEP5**, RFC3, **RFC4**, **SAT**, SLC19A1, **SLC1A4**, SP1, SUZ12, **ZNF655**	17	**12**	Cellular Growth and Proliferation, Cancer, Cell Cycle
	5	ABCB1, AKT1, **APPL**, BCL2L11, **CA12**, CCL21, CCR5, CD4, CD36, **CD82**, CD1D, DIO1, **FXYD5**, **GLRX**,H2-D1, **HARS**, **HAX1**, **HDAC3**, HDAC9, HIF1AN, HLA-DMB,**HLA-E**, **HRASLS3**, HSP90AA2, HTRA2, IFNG, JDP2, MAP3K7IP2, MAPK8, MECP2, **PEA15**, PPP4C, TAPBP, TCL1A, VHL	15	**11**	Infectious Disease, Cancer
	6	ACTB, CCNA2, **CCND3**, CD14, CDC25A, CEBPB, CEBPE, CSF3R, EDN1, EGR2, **EIF4EBP1**, **EPPB9**, EXOSC10, **FOXRED1**, IFNA1, KITLG, **MID1IP1**, **MPHOSPH6**, MSN, MYC, **N-PAC**, NFYC, NPM1, ODC1, **PHACTR1**, **PIP**, **PLCB4**, PLD2, RPL7, RPS20, SLC2A1, **SNRPN**, SPI1, TCF3, TRIM28	15	**11**	Cellular Development, Immune and Lymphatic System Development and Function
**Down-regulated gene analysis**	7	ACTR2, ARHGAP1, **ARL4A**, ARL6IP, ARPC1B, ASGR2, **BNIP2**, CDC42, CDC42EP5, CTNNB1, **CTPS**, ERBB2, **HOXA5**, HTRA1, IGFBP6, **IQGAP1**, ITSN2, **LOXL2**, **NBN**, **NNMT**, **PFN2**, PTN, SEC61A1, **SEC61B**, **SEPT2**, SEPT6, SEPT7, SEPT9, SLC6A2, TGFB1, TP53, TRIP10, **TSC22D1**, **WAS**, WASPIP	23	**14**	Cellular Assembly and Organization, Cellular Function and Maintenance, Cancer
	8	ACP5, **ARD1A**, BET1,**CD44**, CMA1, CST7, **CTSL**, DEFB103A, DEFB4, FN1, GDF5, GUSB, HIF1A, HIF1AN, HIST1H1C, IER2, IL4, **IQGAP1**, MADCAM1, MST1R, **NASP**, NPHS1, NRG1, PIK3CB, **PPM1G**, **SEC22L1**, SERPINB3, **SLC2A1**, **TFPI2**, **TGFBI**, **TGOLN2**, TNF, TNFAIP6, **TSTA3**, ZAP70	21	**13**	Cell-To-Cell Signaling and Interaction, Tissue Development, Cellular Movement
	9	ADM, AKT1, **BAG1**, BCL2L1, **BMP7**, CALCR, CDK7, CPB2, **CRI1**, EEF1A1, **EEF1B2**, **GABPB2**, **GALNT10**, GNAQ, **GNB5**, **HPRT1**, IL1B, OXTR, **PCGF6**, PROCR, PSMA1, PSMA3, **PSMA4**, PSMA6, PSMB2, PSMB3, PSMB6, PSMB9, PSMB10, **RAMP1**, RB1, **SPARC**, **THBD**, TPT1, YAF2	21	**13**	Cellular Growth and Proliferation, Molecular Transport
	10	AR, **ATP1A3**, BCL2, CCND1, CCNE1, CDK2, CDKN1A, **CHMP4A**, CHMP4C, EGF, EGFR, EPO, GH1, **HEXA**, INS, INS1, MAPK3, **ME1**, NCOR2, NR3C1, **NRAS**, PDCD6IP, PPARA, PTK2B, RXRB, SRC, STAT5B, THRB, TP53, TSG101, VPS28, **VPS37B**, VPS37C	8	**6**	Gene Expression, Cell Cycle, Cancer

Up- and down-regulated genes from L235 analyzed using the IPA tool (version 4.0). Among them 74 up- and 52 down-regulated genes were eligible for generating networks and led to the identification respectively of 10 and 11 distinct networks containing both direct and indirect interactions scored by significance. The six up and four down-regulated networks considered as relevant (i.e. score>3) are reported. Genes selected as differentially expressed in [PIP+] versus [PIP−] samples (i.e. Focus genes) are shown in bold. Underlined genes indicate those belonging to multiple networks. The other genes are either absent from the microarray or found not significantly regulated.

Among the selected networks, several up-regulated genes are found associated with a pro-apoptotic function (*BAD*, *CDKN2A*, *PRAME*) [31]–[33] and an inhibition of cell growth and proliferation (*CCND3*, *CDKN2A*, *EFNA1*, *HRASL3* and *PRAME*)[34]–[39]. These results are concordant with the down-regulation of *ARD1*, *CTPS*, *EEF1B2*, *LOXL2*, *NRAS* and *PTN* known to promote cell proliferation [40]–[47]. Only two over-expressed genes, *MMP14* and *HDAC3*, could result in possible conflicting functions leading to enhanced cell proliferation. For instance, *MMP14* was previously shown to enhance proliferation in many types of tumors [48] and *HDAC3* silencing demonstrated to induce cell cycle arrest and apoptosis [49], [50]. Conversely, most down-regulated genes involved in the cell death pathway are linked to anti-apoptotic functions (*BAG1*, *CTSL*, *CTPS*, *PTN*, *LOXL2*) [44], [51]–[54]. Nevertheless, decrease of expression of the pro-apoptotic *BNIP2* gene could support an opposite effect on cell survival [55].

The expression level of genes implicated in cellular adhesion (*CD82*, *EFNA1*, *ITGB6*, *LGALS8*) is found increased in samples overexpressing *PIP* [56]–[61]. In addition, an overexpression of the *PEA-15* gene is observed in the [PIP+] versus the [PIP−] samples. The PEA-15 function was recently related to cell invasion via its ability to bind to ERK1/2 [62]. It has also been shown that *PEA-15* is expressed in normal mammary gland and exhibits a decreased expression in pathologically invasive cancer, suggesting an inverse relationship between *PEA-15* expression and tumor invasion. All considered, observed up-regulations could reinforce cell adhesion and consequently exert a preventive effect on cell motility and metastasis development. Conversely, *FXYD5*, *ICAM1* and *MMP14* overexpression might enhance tumor invasion [63]–[66]. In fact, several studies on breast cancer samples have shown a major role of the protease MMP14 in the invasion process occurring mainly via extracellular matrix remodelling. These discordant observations may reflect differences in gene expression observed between tumor and cell line models resulting from the environmental specificities of tumor cells *in vivo* and the heterogeneous mixture of cells in tumor samples, including immune cells as well as tissue-specific cells, which cannot be reproduced with cells grown in culture plates [67].

The down-regulated gene analysis also highlighted a network linked to cell to cell signalling and cellular movement function, reflecting an impact of these gene modulations on cell motility and invasiveness (network 8, down-regulated genes, Table 7). Within this network, several genes had a function promoting migration and cell invasiveness (*IQGAP1*, *TGFBI*, *CD44*, *CTSL*, *PTN*) [68]–[71]. Accordingly, their down-regulation in [PIP+] versus [PIP−] samples could have a suppressive effect on cell invasion. Similarly, the down-regulated expression of *LOXL2* might prevent tumor progression, as shown by the induction of the epithelial-to-mesenchymal transition process in epithelial cells overexpressing these genes [44].

In summary, this pathway analysis strongly suggests that the majority of gene modulations, occurring in [PIP+] versus [PIP−] cells, may contribute to a reduction of cell proliferation concomitantly with an increase of apoptosis and cell adhesion.

### Visualization of relevant modulations in a master molecular interaction network

**Figure 4:**
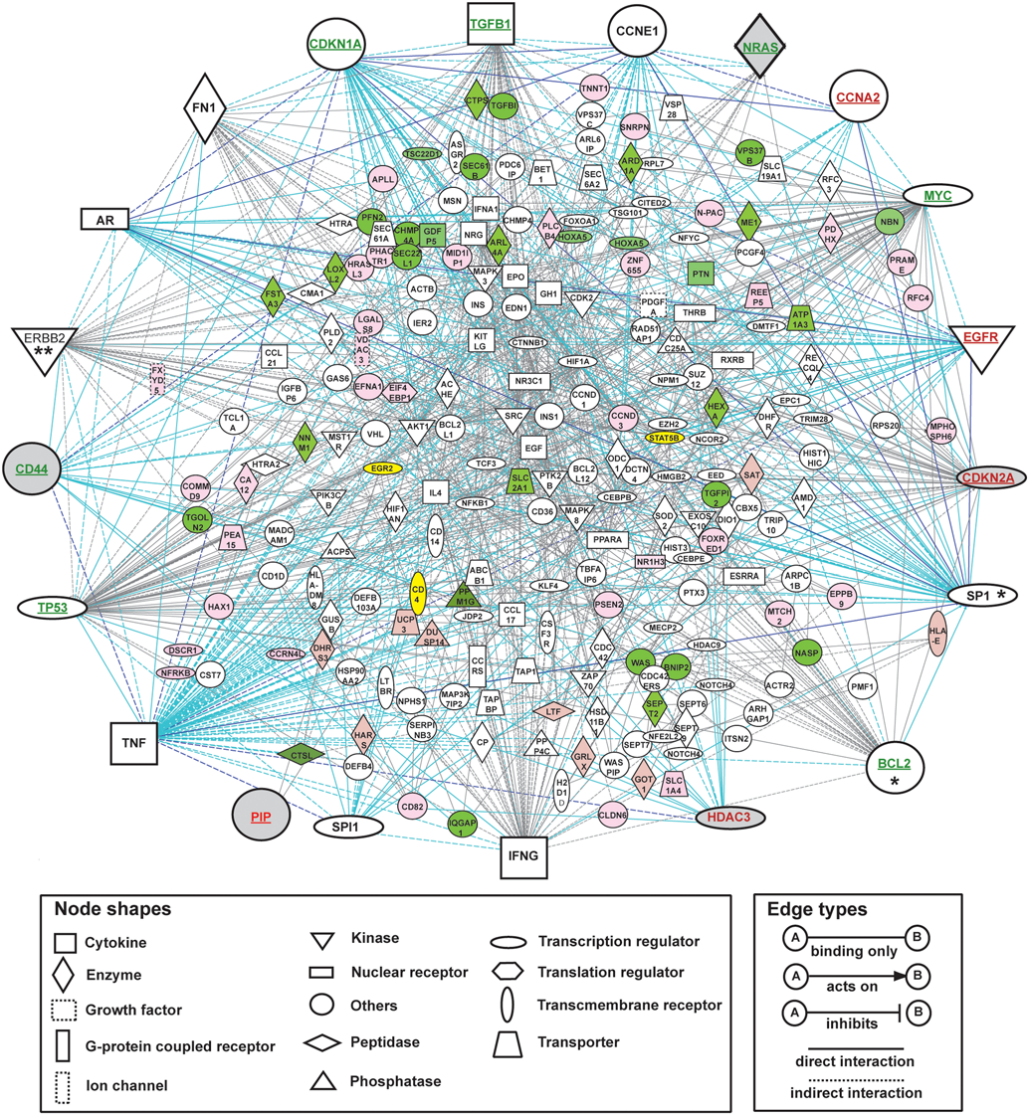
Master molecular network of genes co-modulated with *PIP*. Master network assembled by merging networks 1, 4 & 6 and networks 7, 8 & 10 identified by the IPA tool (version 4.0) from up- and down-regulated gene analysis using overlapping genes (cf. Table 7). The network is displayed graphically as nodes (genes/gene products) and edges (the biological relationship between the nodes). The [PIP+] relative to[PIP−] over-expressed genes are shaded in light red and down-regulated genes in green. The genes connected with *PIP* (*EGR2* and *CD4*) and STAT5B, which was identified by a promoter analysis as a potential key regulator of the master network, are shaded in yellow. The nodes are represented using various shapes that represent the functional class of the gene products. Highly interconnected nodes (‘hub genes’) are moved to the network periphery together with the *PIP* gene. The hub genes belonging to L235 are shaded in gray and those, which were detected by quantitative PCR, are underlined. The gene names are written in green (down-regulated) or in red (up-regulated) relative to a [PIP+] versus [PIP−] modulation. An asterisk refers to a gene that was not selected in L235, but was identified at another level of the statistical analysis (* for L578&L2231 and ** for L1114 & L2231, Table S1).

In order to visualize comprehensive interactions between the modulated genes within breast cancer cells and place them in the context of molecular interaction network, the most significant up- and down-regulated gene networks were merged (Figure 4). Networks 1, 4 and 6 of up-regulated genes were chosen according to the highest score value (23), the corresponding functions in tumorigenesis (proliferation, cancer and cell cycle) and the presence of the *PIP* gene, respectively (Table 7). These networks were merged through the overlapping genes (*CD14* and *TCF3* for networks 1 & 6; *CCND3* for networks 4 & 6). About 45% of the focus genes of those up-regulated gene networks are found to take part in one of the most relevant clusters identified by *t*-statistics in L235 hierarchical clustering. More specifically, 67% of the focus genes from network 1 are included in NODE 222X. Networks 7, 8 and 10 of down-regulated genes were selected upon the presence of overlapping genes between networks: *TP53* for networks 7&10 and *IQGAP1* for networks 7 & 8 (Table 7). An important fraction of the focus genes from those down-regulated gene networks are localized within NODE 167X or NODE 196X from L235 hierarchical clustering.

A 231-member master molecular network has been assembled with 1,262 edges corresponding to a global view of gene expression modulations occurring together with *PIP* gene expression. This master network was constructed by merging the selected up- and down-regulated networks. The nodes and edges for each individual network were added to the merged network together with any new edges that connect these networks, resulting in incorporation of 29 additional genes. Nineteen nodes appeared to be highly connected in the network as demonstrated by the important number of edges emerging from or pointing to them. These nodes were considered as ‘hub genes’ and were moved to the periphery of the network, together with the *PIP* gene, in order to highlight them [72]. Their high connectivity is likely to reflect their ability to regulate an important number of genes within the master network and potentially to control the gene expression modulations identified between cells overexpressing *PIP* or not (Figure 4)[73]. Unexpectedly, of the 15 oncogenes and tumor suppressor genes included in the master network, 10 end up among the 19 hub genes, and 8 of them have been detected as differentially expressed through microarray and/or Q-PCR analyses. Such genes are usually not detected through analysis of differential expression, and are incorporated in predictive network modules only through integration of curated protein-protein interactions [74]. This highlights their central interconnecting role in the master network, and the value of using high-precision expression measurements with careful assessment of statistical power as performed in this study. All 19 hub genes except *AR*, *IFNG*, *SPL1* and *TNF* were present on the array. Among them, *CDKN2A* and *HDAC3* were identified as significantly over-expressed, and *NRAS* and *CD44* as significantly decreased in [PIP+] versus [PIP−] samples, as detected by both microarray analysis (list L235) and Q-PCR (Figure 4, grey shaded symbols and underlined names). Four hub-genes (*EGFR*, *CCNA2*, and *TGFB1*, *TP53* for up- and down-regulated genes, respectively; Figure 4, open symbols and underlined names) were not present in L235 but were found significantly differentially expressed by Q-PCR analysis only; three hub genes (*MYC*, *BCL2*, *CDKN1A*; Figure 4, asterisks) were found significantly differentially expressed by both Q-PCR and microarray analysis, belonging to other relevant computed gene lists. The remaining 4 hub genes (*FN1*, *CCNE1*, *ERBB2*, *SP1*) were not assayed by Q-PCR nor identified as differentially expressed by microarrays except *ERBB2* and *SP1* which were selected in lists L578 and L1114 (See Table S1). As molecular relationships represented on the network include not only induction or inhibition of expression, but also protein-protein interactions, DNA-protein interactions and activation, localization, inhibition of the corresponding proteins, it is not surprising that microarrays and Q-PCR may fail to identify some of the hub genes as being significantly modulated among the [PIP+] and [PIP−] subgroups of samples. These genes might play a major role through protein activation for instance. Alternatively, their modulations may be very subtle and below the threshold for reliable detection of differences of our microarray platform despite its high sensitivity. Distinguishing between these different possibilities will require targeted validation experiments.

The *PIP* gene was also moved to the periphery of the network, even though it is connected to only two other genes, *CD4* and *EGR2* (Figure 4). The edge connecting *PIP* to *EGR2* was previously reported by a microarray study in rat Schwann cells, which demonstrated an up-regulation of *PIP* in cells overexpressing *EGR2* [75]. *EGR2* cDNA clones are represented on the array but no significant modulation of its expression was observed in parallel with the *PIP* gene. The edge connecting *PIP* to *CD4* is based on the reported interaction between these proteins leading to T lymphocyte programmed cell death inhibition induced by CD4 cross-linking and subsequent TCR activation [19]. In our *in vitro* models of breast cancer cell lines, the interaction between the secreted glycoprotein PIP and CD4 cannot take place since CD4 is not expressed in these cells.

In addition, previous studies reported that the PIP protein may exert an aspartyl proteinase activity able to specifically cleave fibronectin (encoded by *FN1*)[20], supporting a link between PIP and FN1 at the protein level. This interaction between *PIP* and *FN1* is actually missing in the IPA database. In spite of this lack, *FN1* appears as a hub gene in our study (Figure 4), thus supporting the strength of the genes identified as involved in the master network associated with *PIP* expression. This indicates that even though more than one million of functional, regulatory and physical interactions are included in the IPA knowledge database, its content is far from being exhaustive. Consequently, other interactions can be missed in the network of the *PIP* co-modulated genes represented in Figure 4.

### Promoter analysis of differentially expressed genes

Co-regulation of mammalian genes usually depends on sets of transcription factors rather than on one individual factor. Therefore an analysis of the promoter regions of the genes from list L235 was conducted in order to identify potential common transcriptional regulators.

Using the cluster ElDoradoe/Gene2promotor/GEMS Launcher for promoter analysis [76], three families of transcription factor binding sites (TFBS) were identified to be common to at least 40% of the genes from L235. They correspond to the glucocorticoid responsive and related elements (GREF), LEF1/TCF (LEFF) and the signal transducer and activator of transcription (STAT). The FrameWorker allowed the identification of a specific promoter framework constituted by all 3 TFBS and shared by 24 gene promoter regions (*ACOT2*, *CEP250*, *GABARAPL1*, *NFRKB*, *NNMT*, *PHACTR1*, *PIP*, *PLCB4*, *PRAME*, *PSEN2*, *PSPH*, *RPN2*, *SAT*, *SC5DL*, *SEPT2*, *SLC1A4*, *SNRPA1*, *SNRPN*, *SNURF*, *SPARC*, *SRPR*, *TCF25*, *TEAD4*, *TGFBI*, *TMED2*). Another set of 26 genes containing only a framework of 2 of the TFBS (GREF-STAT) was identified (*ALG3*, *ANXA9*, *ATP6AP2*, *B4GALT4*, *DHRS3*, *GDF5*, *HAX1*, *HDAC3*, *IQGAP1*, *MPHOSPH6*, *NR1H3*, *PEA-15*, *PFN2*, *RAB13*, *REEP5*, *RFC4*, *SCAMP2*, *SEC22B*, *SLC2A1*, *SUCLA2*, *TFPI2*, *THBD*, *TNNT1*, *TSPAN1*, *TSTA3*, *TULP1*). Specifically, STAT5 transcription factor binding sites were implicated in this GREF-STAT motif. Additionally, a TFBS from the STAT motif family only was identified in the promoter region of several other genes from L235 (data not shown).

In the master network of the differentially expressed genes (Figure 4),a high number of interactions with *STAT5B* is observed (*BCL2*, *CCND1*, *CCR5*, *CDKN1A*, *EGF*, *EPO*, *ERBB2*, *GH1*, *HEXA*, *IFNA1*, *IFNG*, *INS1*, *KITLG*, *NOTCH4*, *PPARA*, *PTK2B*, *TGFB1* and *THRB*). Several of these genes appear to be upstream regulators of *STAT5B*: *EGF*, *EPO*, *IFNA1*, *IFNG* and *PYK2* have been shown to increase the activation of STAT5B [77]–[81] whereas *TGFB1* was shown to increase its expression [82]. In contrast to STAT5B, STAT5A is not represented in the assembled master network (Figure 4). Analysis of known interactions between *STAT5A* and the genes from the master network showed that *STAT5A* share almost the same interactions as *STAT5B* except for *CCND1*, *CCR5*, *ERBB2*, *HEXA*, *PPARA*, *PTK2B*, *TGFB1* and *THRB* (data not shown). Moreover additional interactions have been reported with *BCL2L1*, *EGFR*, *MYC*, *NFKB1*, *NR3C1* and *TNF*. In particular, *TNF* and *NFKB1* have been shown to increase expression of *STAT5A* [83], [84].

Previous studies described that STAT5 may exhibit opposite functions in mammary oncogenesis, either increasing tumor development in several murine models [85]–[87] or inhibiting tumor progression in human breast cancer cells [88], [89]. More recently, it has been proposed that STAT5 may act as a suppressor of invasion, epithelial mesenchymal transition and dispersal of breast cancer cells from the primary tumor [89]–[91]. This was novel in light of the previous tumor-promoting role attributed to STAT5 [89]. The suppressive role of STAT5 on cell invasion was confirmed *in vitro* in the well-differentiated ER-positive breast cancer cells T47D [88]. It was also shown that PRL may suppress human breast cancer cell invasion through multiple mechanisms, such as activation of STAT5 [90]. Indeed, STAT5, one of the main downstream effector molecules of PRL [90], has been shown to directly modulate transcriptional activity through interaction with the promoter region of the target genes [92]. Moreover, high levels of activated STAT5 have been found in a substantial proportion of human breast tumors, which interestingly exhibited a better prognosis [88], [93].

Interestingly, *PIP* gene expression was previously reported to be synergistically induced by prolactin (PRL)-activated STAT5 and DHT-activated AR. More precisely PRL-induced phosphorylation on Tyr694 of STAT5A and Tyr699 of STAT5B was demonstrated to be required for the synergistic effect of DHT and PRL on transcriptional activation of the *PIP/GCDFP-15* gene [94].

Altogether, our results suggest the potential involvement of *STAT5* in the transcriptional regulation of several genes from the master network identified (Figure 4) associated with *PIP*. The failure to detect significant expression changes of *STAT5A* and *STAT5B* genes in *PIP* expressing versus non expressing breast carcinoma cells using microarray analysis suggests that the protective effects of these transcription factors on breast carcinoma development could be mainly due to their activation rather than to modifications of their gene expression levels. This hypothesis, supported by the suppression of cell invasion through STAT5 activation [90], will have to be further investigated in future studies.

In summary, we report here a comprehensive characterization of the gene expression modulations occurring in *PIP*-expressing versus non-expressing breast cancer cell lines. Using rigorous unsupervised and supervised analyses, we identified differentially expressed genes, which were found strictly co-modulated in relation to the *PIP* expression level changes and allowed us to discriminate [PIP+] and [PIP−] subgroups of samples. This study provides useful information in term of pathway modulations that occur within breast cells expressing *PIP*. The combination of a high-precision expression profiling with an extensive functional and regulatory network analysis has emphasized a central interconnecting role of a number of oncogenes and tumor suppressor genes in the network associated with *PIP* expression modulation. Many oncogenes and tumor suppressor genes, previously reported to exhibit particular breast cancer mutations, e.g. *ERBB2* and *TP53*, are typically not detected through analysis of differential expression but can play a central role in signalling networks by interconnecting many expression-responsive genes [74]. Interestingly, half of them were found significantly differentially expressed with an increase level of PIP transcript. Consequently, our data allowed determination of a global view of the regulatory network resulting from *PIP* overexpression based on the aggregate behaviour of genes connected in a functional network rather than on unique genes found differentially expressed.

Functionally, the gene expression modulations associated with an increase of levels of *PIP* transcript appear associated with an inhibition of proliferation coupled with an enhancement of the apoptosis and the cell adhesion in breast cancer cell lines. These results provide additional and contextual support for the good prognostic value of *PIP* gene expression in breast cancer, as recently demonstrated by immunohistochemistry on a large cohort of tumor samples in which significantly longer disease-free survival times were associated with PIP positive tumors [95]. In addition, STAT5 was identified through *in silico* promoter analyses of the genes co-modulated with *PIP* suggesting that it might be a transcriptional regulator accounting for the observed altered functions. This unexpected result supports the view that an important part of the modulated genes act as upstream or downstream effectors of STAT5. For some of them, there is no experimental evidence of a relationship with STAT5 and additional experiments would be required to confirm this point. Finally, STAT5 is known to inhibit cell invasion and is considered as a good prognostic factor in breast cancer [88], [89].

Many of the groups of genes (Table 7) that form the basis of the master network reported here (Figure 4), including those discussed above, represent novel combinations of factors that may impact on important cancer-initiating biological processes or that may be modulated consequentially. Further biological and clinical investigations using a large cohort of patients will be necessary to identify those which contribute directly to breast cancer development and progression, have prognostic value and are possible targets for therapeutic intervention.

## Materials and Methods

### Cell lines and culture conditions

T47D, MCF7, MDA-MB231 breast carcinoma cell lines were obtained from the American Type Culture Collection. VHB1 cells [96] were a gift from J. Soudon (Hopital Saint-Louis, Paris, France). Cells were maintained in Dulbecco's modified Eagle's medium (DMEM) with GlutaMAX (Invitrogen Ltd., Paisley, UK), supplemented with 10% foetal calf serum (Perbio Sciences, Helsingborg, Sweden), 100 U/ml penicillin, 100 µg/ml streptomycin in a 5% CO_2_ incubator. Cells were treated with 10 nM Dihydrotestosterone (DHT; Sigma #A8380, St Louis, MO) for 6, 7, 8 or 10 days before RNA extraction.

### RNA extraction

Total RNA was extracted from monolayer cells in culture at 2/3 confluency using the RNeasy Mini Kit (Qiagen, Hilden, Germany). RNA purity and quantity was assessed by UV measurement. Healthy mammary gland RNA from three distinct healthy donors and a universal human reference RNA were obtained from commercial sources (Stratagene Europe, Amsterdam, Netherlands). RNA integrity was judged using RNA 6000 nano chips and the Agilent 2100 Bioanalyzer (Agilent Technologies, Palo Alto, CA) according to the manufacturer's instructions. RNA quality-control was performed using user-independent classifiers as described [97], [98].

### Northern blot analysis

Total RNAs (15–50 µg) from breast carcinoma cell lines were electrophoresed in a 1.6% formaldehyde agarose gel and transferred onto Hybond-N nylon membranes (Amersham Biosciences, Buckinghamshire, UK) according to standard techniques [99]. Probes labeled with [α-^32^P] dCTP (3000 Ci/mmol; Amersham Biosciences) were full-length PIP and ß-actin cDNAs [100], [101]. Northern blots were hybridized at 68°C for 16 h with ^32^P-labeled probes (1.5×10^6^ cpm/ml) in ExpressHyb Hybridization solution (BD Biosciences Clontech), washed twice in 2× SSC/0.05% SDS at room temperature for 30 min and twice in 0.1× SSC/0.1% SDS at 50°C for 45 min. Membranes were then autoradiographed at −80°C on Kodak X-Omat AR X-ray films (Kodak, Rochester, NY).

### Microarray design and manufacture

The human cDNA microarrays used contained 11,520 sequences derived from various sequence-verified clone collections as previously described [102]. The array set provides a genome-wide coverage of functional pathways, such as cell cycle and checkpoints, cell growth and/or maintenance, cell adhesion and proliferation, development, extracellular matrix, apoptosis, response to DNA damage and DNA repair, DNA replication, transcription and RNA processing. High confidence qualifications and annotations of the clone collections have been previously described [102] and are available through our web site (The Genexpress - Array s/IMAGE web site,[103]). All arrays were printed in the laboratory on amino-modified mirrored glass slides using the Lucidea array spotter (Amersham Biosciences) as described [102]. The suite of amplified cDNAs was printed as a group in two spatially separated replicates.

### Hybridization experimental design and analysis

To reduce potential experimental biases, four independent RNA preparations were collected for each DHT-treated and -untreated cell lines. To assess data reproducibility and minimize dye bias effects, each of the samples was measured twice, once with Cy3 and once with Cy5. To ensure robustness and flexibility in data analysis, a reference design was used with a universal reference sample (Stratagene) serving as a baseline for the comparisons of cell line samples. Such a design does not require pre-definition of the subgroups for comparison, allows robust discovery of non-anticipated classes among the samples and is compatible with subsequent additional sampling [102].

Thirty µg of total RNA from each cell line and human universal reference RNA (Stratagene) were supplemented with known sequences (spikes, Universal ScoreCard), reverse transcribed using an oligo-dT primer and labeled alternatively with Cy-5-dCTP and Cy-3-dCTP (Amersham Biosciences). Samples were purified using the Qiagen's QIAquick PCR Purification kit procedure and submitted to a vigilant quality control procedure as previously described [102]. Hybridizations to the arrays were performed as described [102]. Array images and raw data were obtained using the GenIII array scanner (Amersham Biosciences) and ArrayVision 7.0 software (Imaging Research Inc., Amersham Biosciences, Palo Alto, CA, USA). Raw data were first imported into a Genetraffic duo database (Iobion Informatics, Toronto, Canada), local background-subtracted and normalized using a Lowess (locally weighted linear regression) transformation. The following selection criteria were applied: all spots having a mean signal (after background subtraction) less than that of the background and below that of the negative controls in both Cy3 and Cy5 channels were systematically excluded; the data were also filtered to exclude spots flagged as missing or corrupted in one array. For arrays considered as partially exploitable based on several quality criteria additional hybridizations were done and considered as technical replicates. We next calculated the average expression ratios (test/reference) in all analyses. Log2 values of lowess-transformed data were used for all subsequent statistical analyses. For reporting genes by name, IMAGE Clone IDs corresponding to the microarray probe sequences were used to extract UniGene Cluster IDs and names (Build 199 Homo sapiens; Jan 16 2007)[104]. For genes represented by multiple probes (that is, different clones corresponding to the same gene) on the array, each probe and the related expression ratios were considered and reported separately. MIAME-compliant data [105] have been deposited in the Gene Expression Omnibus (GEO) at NCBI [106] and are accessible through GEO Series accession number GSE11627.

### Modeling of experimental power

For statistical confidence and power analyses related to this specific program, power (z-score) for an unpaired t-test (two-sample analyses) was computed as previously described [102] for estimation of false discoveries (FDR) [107] and using the GPower3.0.3 program [108], [109] for estimation of false negatives (FNR), taking into account the standard deviation of expression measurements, the size of the distinct sample groups, a significance threshold and the fold ratio to be detected.

*A priori* power analyses were used to choose the appropriate number of replicates before the study was conducted. Conversely, *post hoc* power calculations were done to evaluate the actual power reached in our study.

### Hierarchical clustering

For discriminant analysis of overall variation in samples/genes, median centering and normalization of the genes and samples were applied to the entire dataset. Genes which had missing values in more than 20% of the samples were removed from subsequent analysis. An unsupervised average-linkage hierarchical clustering algorithm using a centered Pearson correlation as similarity metric was applied to investigate relationships between samples and relationships between genes. This method leads to an expression matrix such that genes and samples with similar expression patterns are adjacent to each other. This analysis was performed using Cluster [110] and the resulting expression map was visualized with TreeView [110].

For discriminant analysis of differentially expressed genes, an average-linkage hierarchical clustering with uncentered Pearson correlation was applied to the dataset extracted from the list of the genes selected to be differentially expressed. Mean sample profiles and gene profiles were ranked based on a discrimination score, which is equivalent to the *t*-statistics z-score, using the Cluster Identification Tool (CIT), based on supervised *t*-statistics with permutation [111]. Discriminant analysis of the [PIP+] and [PIP−] samples was performed, providing a list of gene nodes that exhibit statistically significant differential expression between the two groups (α≤0.05).

### Statistical analysis

The statistical significance of measured intensity differences was tested using ArrayStat 1.0 software (Imaging Research Inc.). The whole data sets were adjusted using additive statistical models considering samples with homogeneous phenotypes ([PIP++], [PIP+] or [PIP−]) as replicates measures from one condition, with a minimum of 67–75% registered measures per gene. Offset corrections were applied to compensate any potential systematic errors that may exist within data for each condition across arrays. Random error was estimated using a curve-fit method; outliers were automatically detected and then excluded from subsequent analysis, based on thresholds computed over the entire dataset (in median absolute deviation (MAD) and standard deviation (sd) units). For [PIP+] vs [PIP−], [PIP+] J = 0 vs [PIP−] J = 0, [PIP+] J = 7 vs [PIP−] J = 7 and J = 0 vs J = 7 comparisons, data sets were centered for each condition and *t*-, Z-statistics and *F*-statistics were applied with false discovery rate (FDR) corrections to compensate for multiple testing effects [112]. Data from genes with significant differential expression levels between the two compared subgroups were displayed, together with a two-tailed *p*-value adjusted with α = 0.01.

Independent statistical analysis was achieved using SAM (Significant Analysis of Microarrays, Standford University)[113]. This class comparison method uses a modified t-test to identify genes that discriminate, for example, [PIP+] samples from [PIP−] samples. The modified *t*-test involves carrying out typical *t*-tests for each gene using the original data and a user-specified number of permuted datasets generated by randomly shuffling of the class labels. We conducted SAM analysis based on the microarray intensity level (in arbitrary units, A.U.) of the *PIP* gene in samples (T47D J = 0∶20 000 A.U.; T47D J = 7∶30 000 A.U.; VHB1 J = 0∶800 A.U.; VHB1 J = 7∶7 000 A.U.; MCF7 J = 0 and J = 7∶800 A.U.; MDA-MB231 J = 0∶1 000 A.U.; MDA-MB231 J = 7∶1 500 A.U.) using a false positive rate of 0.01 and a number of permutations of 2 000.

### Real-time Quantitative RT-PCR (Q-PCR) analysis using Taqman Low Density Arrays

Pre-defined TaqMan probe and primer sets for target genes were chosen from an on-line database (Applied Biosystems, Foster City, CA,[114]). The sets were factory-loaded into 384 well microfluidic cards (Applied Biosystems) as customized with two replicates per target gene. Single-stranded cDNA was prepared from 1 µg of total RNA from breast carcinoma cell lines using the high capacity cDNA archive kit (Applied Biosystems), according to the manufacturer's instructions. Breast carcinoma cell line RNA samples derived from identical preparations for both cDNA microarray and Q-PCR analysis.

Two µl of single-stranded cDNA (equivalent to 100 ng of total RNA) were mixed with 48 µl of nuclease-free water and 50 µl of TaqMan Universal PCR Master Mix (Applied Biosystems). The sample-specific PCR mixture (100 µl) was loaded into one sample port, the cards were centrifuged twice for 1 min at 280 *g* and sealed to prevent well-to-well contamination. The cards were placed in the Micro Fluidic Card Sample Block of an ABI Prism 7900 HT Sequence Detection System (Applied Biosystems). The thermal cycling conditions were 2 min at 50 °C and 10 min at 95 °C, followed by 40 cycles of 30 s at 97 °C and 1 min at 59.7 °C. 96 genes were tested by quantitative PCR, using the TaqMan low density micro fluidic card (Applied Biosystems, USA). Raw data are available upon request.

### Network and Gene Ontology analysis

The differentially expressed genes were used for pathway and Gene Ontology analyses. Locuslink ID gene accession numbers and their corresponding fold changes in our experiment were imported into the Ingenuity Pathway Analysis (IPA) tool and mapped to its corresponding gene object in the Ingenuity Pathways Knowledge Base (Ingenuity Systems,[115]). Genes were categorized based on their molecular functions using the software, mapped onto genetic networks in the IPA database and then ranked by score. The score associated with a particular network is the likehood (i.e. negative log of a *p*-value) of the genes identified as differentially expressed in a network being found together due to chance. The score is thus indicative of the proportion of genes identified as differentially expressed in our analysis among all the genes belonging to a particular network. A score of 3 reflects the likelihood that the presence of the focus genes in a network is solely due to chance is 1/1000. Therefore, scores of 3 or higher represent a >99.9% confidence level. Genes and gene products are represented as nodes and the biological relationship between two nodes is represented as an edge (line).

In functional analyses, the biological functions that were most significant to the dataset were identified. The significance value assigned to the functions is calculated using the one-side right-tailed Fisher's Exact Test (α = 0.05) of the IPA tool. In this statistical test, the chances that the genes-of-interest participate in the biological functions are examined. A *p*-value is calculated by comparing the number of genes-of-interest in a particular function with their occurrences in all the functions in the IPA knowledge database.

### Promoter sequence analysis

The human promoter sequences for all genes from L235 were extracted with the ElDoradoe/Gene2promotor system ([76]; default 500-bp upstream of the transcription start site and 100-bp downstream). The GEMS Launcher software was used to search for common transcription factor binding sites (TFBSs) in multiple sequences. The quorum constraint which determines the lower limit of loci within the input set that has to contain the common framework was set to 40% (core similarity 1). The selection of matrices associated with specific tissue was restricted to breast tissue.

The FrameWorker task of GEMS Launcher package [116] was then used to retrieve common motifs (frameworks) of transcription factor binding sites in the promoter region of the input genes.

## Supporting Information

Table S1 Origins of gene lists derived from class comparison and class prediction of relative expression levels. Differential expression analyses were conducted to identify genes co-modulated with PIP using several statistical t-, z- and F-tests and an Î± of 0.01. These analyses were conducted initially with a two-phenotype sample classification [PIP−] and [PIP+] and further partitioning the [PIP+] group in two subgroups, in order to anticipate potential genes co-regulation in relation with the PIP expression. The list L219 represents the intersection (∩) of several gene lists (L606, L964, L1114 and L1184) whereas L2231 correspond to the union (∪) of these lists. * Quantitative analysis was based on the microarray intensity level (in arbitrary units, A.U.) of the PIP gene in samples: T47D J = 0∶20 000 A.U.; T47D J = 7∶30 000 A.U.; VHB1 J = 0∶800 A.U.; VHB1 J = 7∶7 000 A.U.; MCF7 J = 0 and J = 7∶800 A.U.; MDA-MB231 J = 0∶1 000 A.U.; MDA-MB231 J = 7∶1 500 A.U.
(0.02 MB XLS)

Table S2 Descriptive statistics and annotation of differentially expressed genes from L235.
(0.21 MB XLS)

## References

[1] McPherson K, Steel CM, Dixon JM (1994). ABC of breast diseases. Breast cancer epidemiology, risk factors and genetics.. BMJ.

[2] Harbeck N, Dettmar P, Thomssen C, Berger U, Ulm K (1999). Risk-group discrimination in node-negative breast cancer using invasion and proliferation markers: 6-year median follow-up.. Br J Cancer.

[3] Brenner AJ, Aldaz CM (1997). The genetics of sporadic breast cancer.. Prog Clin Biol Res.

[4] Wells CA, McGregor IL, Makunura CN, Yeomans P, Davies JD (1995). Apocrine adenosis: a precursor of aggressive breast cancer?. J Clin Pathol.

[5] Wick MR, Lillemoe TJ, Copland GT, Swanson PE, Manivel JC (1989). Gross cystic disease fluid protein-15 as a marker for breast cancer: immunohistochemical analysis of 690 human neoplasms and comparison with alpha-lactalbumin.. Hum Pathol.

[6] Moritani S, Ichihara S, Hasegawa M, Endo T, Oiwa M (2008). Serous papillary adenocarcinoma of the female genital organs and invasive micropapillary carcinoma of the breast. Are WT1, CA125, and GCDFP-15 useful in differential diagnosis?. Hum Pathol.

[7] Fiel MI, Cernaianu G, Burstein DE, Batheja N (1996). Value of GCDFP-15 (BRST-2) as a specific immunocytochemical marker for breast carcinoma in cytologic specimens.. Acta Cytol.

[8] Zagorianakou P, Zagorianakou N, Stefanou D, Makrydimas G, Agnantis NJ (2006). The enigmatic nature of apocrine breast lesions.. Virchows Arch.

[9] Perry A, Parisi JE, Kurtin PJ (1997). Metastatic adenocarcinoma to the brain: an immunohistochemical approach.. Hum Pathol.

[10] Akasofu M, Kawahara E, Kurumaya H, Nakanishi I (1993). Immunohistochemical detection of breast specific antigens and cytokeratins in metastatic breast carcinoma in the liver.. Acta Pathol Jpn.

[11] Monteagudo C, Merino MJ, LaPorte N, Neumann RD (1991). Value of gross cystic disease fluid protein-15 in distinguishing metastatic breast carcinomas among poorly differentiated neoplasms involving the ovary.. Hum Pathol.

[12] Park SY, Kim BH, Kim JH, Lee S, Kang GH (2007). Panels of immunohistochemical markers help determine primary sites of metastatic adenocarcinoma.. Arch Pathol Lab Med.

[13] Murphy LC, Lee-Wing M, Goldenberg GJ, Shiu RPC (1987). Expression of the gene encoding a prolactin-inducible protein in human breast cancers in vivo: a correlation with steroid receptor status.. Cancer Res.

[14] Clark JW, Snell L, Shiu RP, Orr FW, Maitre N (1999). The potential role for prolactin-inducible protein (PIP) as a marker of human breast cancer micrometastasis.. Br J Cancer.

[15] Haagensen JDE, Mazoujian G, Haagensen CD (1986). Biochemistry and immunochemistry of gross cystic disease fluid proteins of the breast.. Diseases of the Breast. 3rd ed.

[16] Autiero M, Abrescia P, Guardiola J (1991). Interaction of seminal plasma proteins with cell surface antigens: presence of a CD4-binding glycoprotein in human seminal plasma.. Exp Cell Res.

[17] Autiero M, Cammarota G, Friedlein A, Zulauf M, Chiappetta G (1995). A 17 kDa CD4-binding glycoprotein present in human seminal plasma and in breast tumor cells.. Eur J Immunol.

[18] Basmaciogullari S, Autiero M, Culerrier R, Mani JC, Gaubin M (2000). Mapping the interaction site between CD4 and gp17, a glycoprotein secreted from seminal vesicles and breast carcinomas.. Biochemistry.

[19] Gaubin M, Autiero M, Basmaciogullari S, Métivier D, Mishal Z (1999). Potent inhibition of CD4/TCR-mediated T cell apoptosis by a CD4-binding glycoprotein secreted from breast tumor and seminal vesicle cells.. J Immunol.

[20] Caputo E, Manco G, Mandrich L, Guardiola J (2000). A novel aspartyl proteinase from apocrine epithelia and breast tumors.. J Biol Chem.

[21] Lander ES, Linton LM, Birren B, Nusbaum C, Zody MC (2001). Initial sequencing and analysis of the human genome.. Nature.

[22] Autiero M, Culerrier R, Bouchier C, Basmaciogullari S, Gaubin M (1999). Abnormal restriction pattern of PIPgene associated with human primary prostate cancers.. DNA Cell Biol.

[23] Autiero M, Camarca A, Ciullo M, Debily MA, El Marhomy S (2002). Intragenic amplification and formation of extrachromosomal small circular DNA molecules from the PIP gene on chromosome 7 in primary breast carcinomas.. Int J Cancer.

[24] Ciullo M, Debily MA, Rozier L, Autiero M, Billault A (2002). Initiation of the breakage-fusion-bridge mechanism through common fragile site activation in human breast cancer cells: the model of *PIP* gene duplication from a break at FRA7I.. Hum Mol Genet.

[25] Thompson EW, Paik S, Brunner N, Sommers CL, Zugmaier G (1992). Association of increased basement membrane invasiveness with absence of estrogen receptor and expression of vimentin in human breast cancer cell lines.. J Cell Physiol.

[26] Loos S, Schulz KD, Hackenberg R (1999). Regulation of GCDFP-15 expression in human mammary cancer cells.. Int J Mol Med.

[27] Sorlie T, Perou CM, Tibshirani R, Aas T, Geisler S (2001). Gene expression patterns of breast carcinomas distinguish tumor subclasses with clinical implications.. Proc Natl Acad Sci U S A.

[28] Simard J, Hatton AC, Labrie C, Dauvois S, Zhao HF (1989). Inhibitory effect of estrogens on GCDFP-15 mRNA levels and secretion in ZR-75-1 human breast cancer cells.. Mol Endocrinol.

[29] ([http://genome-www5.stanford.edu/cgibin/source/sourceSearch]) The SOURCE browser

[30] Livak KJ, Schmittgen TD (2001). Analysis of relative gene expression data using real-time quantitative PCR and the 2(-Delta Delta C(T)) Method.. Methods.

[31] Chao DT, Korsmeyer SJ (1998). BCL-2 family: regulators of cell death.. Annu Rev Immunol.

[32] Jin X, Nguyen D, Zhang WW, Kyritsis AP, Roth JA (1995). Cell cycle arrest and inhibition of tumor cell proliferation by the p16INK4 gene mediated by an adenovirus vector.. Cancer Res.

[33] Campbell I, Magliocco A, Moyana T, Zheng C, Xiang J (2000). Adenovirus-mediated p16INK4 gene transfer significantly suppresses human breast cancer growth.. Cancer Gene Ther.

[34] Sers C, Emmenegger U, Husmann K, Bucher K, Andres AC (1997). Growth-inhibitory activity and downregulation of the class II tumor-suppressor gene H-rev107 in tumor cell lines and experimental tumors.. J Cell Biol.

[35] Tajeddine N, Gala JL, Louis M, Van Schoor M, Tombal B (2005). Tumor-associated antigen preferentially expressed antigen of melanoma (PRAME) induces caspase-independent cell death in vitro and reduces tumorigenicity in vivo.. Cancer Res.

[36] Fox BP, Kandpal RP (2004). Invasiveness of breast carcinoma cells and transcript profile: Eph receptors and ephrin ligands as molecular markers of potential diagnostic and prognostic application.. Biochem Biophys Res Commun.

[37] Rhee K, Bresnahan W, Hirai A, Hirai M, Thompson EA (1995). c-Myc and cyclin D3 (CcnD3) genes are independent targets for glucocorticoid inhibition of lymphoid cell proliferation.. Cancer Res.

[38] Duan J, Chen Z, Liu P, Zhang Z, Tong T (2004). Wild-type p16INK4a suppresses cell growth, telomerase activity and DNA repair in human breast cancer MCF-7 cells.. Int J Oncol.

[39] Parri M, Buricchi F, Taddei ML, Giannoni E, Raugei G (2005). EphrinA1 repulsive response is regulated by an EphA2 tyrosine phosphatase.. J Biol Chem.

[40] Cho DI, Oak MH, Yang HJ, Choi HK, Janssen GM (2003). Direct and biochemical interaction between dopamine D3 receptor and elongation factor-1Bbetagamma.. Life Sci.

[41] Le Sourd F, Boulben S, Le Bouffant R, Cormier P, Morales J (2006). eEF1B: At the dawn of the 21st century.. Biochim Biophys Acta.

[42] Lim JH, Park JW, Chun YS (2006). Human arrest defective 1 acetylates and activates beta-catenin, promoting lung cancer cell proliferation.. Cancer Res.

[43] Agbaria R, Kelley JA, Jackman J, Viola JJ, Ram Z (1997). Antiproliferative effects of cyclopentenyl cytosine (NSC 375575) in human glioblastoma cells.. Oncol Res.

[44] Peinado H, Del Carmen Iglesias-de la Cruz M, Olmeda D, Csiszar K, Fong KS (2005). A molecular role for lysyl oxidase-like 2 enzyme in snail regulation and tumor progression.. Embo J.

[45] Riegel AT, Wellstein A (1994). The potential role of the heparin-binding growth factor pleiotrophin in breast cancer.. Breast Cancer Res Treat.

[46] Li T, Sparano JA (2003). Inhibiting Ras signaling in the therapy of breast cancer.. Clin Breast Cancer.

[47] Garbay C, Liu WQ, Vidal M, Roques BP (2000). Inhibitors of Ras signal transduction as antitumor agents.. Biochem Pharmacol.

[48] Tetu B, Brisson J, Wang CS, Lapointe H, Beaudry G (2006). The influence of MMP-14, TIMP-2 and MMP-2 expression on breast cancer prognosis.. Breast Cancer Res.

[49] Huang W, Tan D, Wang X, Han S, Tan J (2006). Histone deacetylase 3 represses p15(INK4b) and p21(WAF1/cip1) transcription by interacting with Sp1.. Biochem Biophys Res Commun.

[50] Glaser KB, Li J, Staver MJ, Wei RQ, Albert DH (2003). Role of class I and class II histone deacetylases in carcinoma cells using siRNA.. Biochem Biophys Res Commun.

[51] Bowden ET, Stoica GE, Wellstein A (2002). Anti-apoptotic signaling of pleiotrophin through its receptor, anaplastic lymphoma kinase.. J Biol Chem.

[52] Kim K, Cai J, Shuja S, Kuo T, Murnane MJ (1998). Presence of activated ras correlates with increased cysteine proteinase activities in human colorectal carcinomas.. Int J Cancer.

[53] Kudoh M, Knee DA, Takayama S, Reed JC (2002). Bag1 proteins regulate growth and survival of ZR-75-1 human breast cancer cells.. Cancer Res.

[54] Tobin DJ, Foitzik K, Reinheckel T, Mecklenburg L, Botchkarev VA (2002). The lysosomal protease cathepsin L is an important regulator of keratinocyte and melanocyte differentiation during hair follicle morphogenesis and cycling.. Am J Pathol.

[55] Qin W, Hu J, Guo M, Xu J, Li J (2003). BNIPL-2, a novel homologue of BNIP-2, interacts with Bcl-2 and Cdc42GAP in apoptosis.. Biochem Biophys Res Commun.

[56] Yang X, Wei LL, Tang C, Slack R, Mueller S (2001). Overexpression of KAI1 suppresses in vitro invasiveness and in vivo metastasis in breast cancer cells.. Cancer Res.

[57] Liu WM, Zhang XA (2006). KAI1/CD82, a tumor metastasis suppressor.. Cancer Lett.

[58] Nagy N, Bronckart Y, Camby I, Legendre H, Lahm H (2002). Galectin-8 expression decreases in cancer compared with normal and dysplastic human colon tissue and acts significantly on human colon cancer cell migration as a suppressor.. Gut.

[59] Wang CH, Chang HC, Hung WC (2006). p16 inhibits matrix metalloproteinase-2 expression via suppression of Sp1-mediated gene transcription.. J Cell Physiol.

[60] Miao H, Burnett E, Kinch M, Simon E, Wang B (2000). Activation of EphA2 kinase suppresses integrin function and causes focal-adhesion-kinase dephosphorylation.. Nat Cell Biol.

[61] Mogi S, Dang D, Van Waes C, Ellis D, Atakilit A (2005). The expression of integrin alpha(v)beta6 promotes the epithelial cell morphology and suppresses invasive behavior in transformed oral keratinocytes.. Anticancer Res.

[62] Glading A, Koziol JA, Krueger J, Ginsberg MH (2007). PEA-15 Inhibits Tumor Cell Invasion by Binding to Extracellular Signal-Regulated Kinase 1/2.. Cancer Res.

[63] Mylona E, Nomikos A, Magkou C, Kamberou M, Papassideri I (2007). The clinicopathological and prognostic significance of membrane type 1 matrix metalloproteinase (MT1-MMP) and MMP-9 according to their localization in invasive breast carcinoma.. Histopathology.

[64] Kim HJ, Park CI, Park BW, Lee HD, Jung WH (2006). Expression of MT-1 MMP, MMP2, MMP9 and TIMP2 mRNAs in ductal carcinoma in situ and invasive ductal carcinoma of the breast.. Yonsei Med J.

[65] Rosette C, Roth RB, Oeth P, Braun A, Kammerer S (2005). Role of ICAM1 in invasion of human breast cancer cells.. Carcinogenesis.

[66] Nam JS, Kang MJ, Suchar AM, Shimamura T, Kohn EA (2006). Chemokine (C-C motif) ligand 2 mediates the prometastatic effect of dysadherin in human breast cancer cells.. Cancer Res.

[67] Sandberg R, Ernberg I (2005). The molecular portrait of in vitro growth by meta-analysis of gene-expression profiles.. Genome Biol.

[68] Lyzak JS, Yaremko ML, Recant W, Baunoch DA, Joseph L (1997). Role of CD44 in nonpalpable T1a and T1b breast cancer.. Hum Pathol.

[69] Joensuu H, Klemi PJ, Toikkanen S, Jalkanen S (1993). Glycoprotein CD44 expression and its association with survival in breast cancer.. Am J Pathol.

[70] Yasuda M, Tanaka Y, Fujii K, Yasumoto K (2001). CD44 stimulation down-regulates Fas expression and Fas-mediated apoptosis of lung cancer cells.. Int Immunol.

[71] Niedergethmann M, Hildenbrand R, Wolf G, Verbeke CS, Richter A (2000). Angiogenesis and cathepsin expression are prognostic factors in pancreatic adenocarcinoma after curative resection.. Int J Pancreatol.

[72] Barabasi AL, Oltvai ZN (2004). Network biology: understanding the cell's functional organization.. Nat Rev Genet.

[73] Benson M, Breitling R (2006). Network theory to understand microarray studies of complex diseases.. Curr Mol Med.

[74] Chuang HY, Lee E, Liu YT, Lee D, Ideker T (2007). Network-based classification of breast cancer metastasis.. Mol Syst Biol.

[75] Nagarajan R, Svaren J, Le N, Araki T, Watson M (2001). EGR2 mutations in inherited neuropathies dominant-negatively inhibit myelin gene expression.. Neuron.

[76] ([http://www.genomatix.de/products/ElDorado/index.html]) ElDoradoe/Gene2promotor system

[77] Kloth MT, Catling AD, Silva CM (2002). Novel activation of STAT5b in response to epidermal growth factor.. J Biol Chem.

[78] Cull VS, Tilbrook PA, Bartlett EJ, Brekalo NL, James CM (2003). Type I interferon differential therapy for erythroleukemia: specificity of STAT activation.. Blood.

[79] Leonard WJ, O'Shea JJ (1998). Jaks and STATs: biological implications.. Annu Rev Immunol.

[80] Meyer AN, Gastwirt RF, Schlaepfer DD, Donoghue DJ (2004). The cytoplasmic tyrosine kinase Pyk2 as a novel effector of fibroblast growth factor receptor 3 activation.. J Biol Chem.

[81] Hwa V, Little B, Kofoed EM, Rosenfeld RG (2004). Transcriptional regulation of insulin-like growth factor-I by interferon-gamma requires STAT-5b.. J Biol Chem.

[82] Brizzi MF, Dentelli P, Rosso A, Calvi C, Gambino R (2004). RAGE- and TGF-beta receptor-mediated signals converge on STAT5 and p21waf to control cell-cycle progression of mesangial cells: a possible role in the development and progression of diabetic nephropathy.. Faseb J.

[83] Banno T, Gazel A, Blumenberg M (2004). Effects of tumor necrosis factor-alpha (TNF alpha) in epidermal keratinocytes revealed using global transcriptional profiling.. J Biol Chem.

[84] Hinata K, Gervin AM, Jennifer Zhang Y, Khavari PA (2003). Divergent gene regulation and growth effects by NF-kappa B in epithelial and mesenchymal cells of human skin.. Oncogene.

[85] Humphreys RC, Hennighausen L (1999). Signal transducer and activator of transcription 5a influences mammary epithelial cell survival and tumorigenesis.. Cell Growth Differ.

[86] Iavnilovitch E, Groner B, Barash I (2002). Overexpression and forced activation of stat5 in mammary gland of transgenic mice promotes cellular proliferation, enhances differentiation, and delays postlactational apoptosis.. Mol Cancer Res.

[87] Bertucci F, Nasser V, Granjeaud S, Eisinger F, Adelaide J (2002). Gene expression profiles of poor-prognosis primary breast cancer correlate with survival.. Hum Mol Genet.

[88] Nevalainen MT, Xie J, Torhorst J, Bubendorf L, Haas P (2004). Signal transducer and activator of transcription-5 activation and breast cancer prognosis.. J Clin Oncol.

[89] Sultan AS, Xie J, LeBaron MJ, Ealley EL, Nevalainen MT (2005). Stat5 promotes homotypic adhesion and inhibits invasive characteristics of human breast cancer cells.. Oncogene.

[90] Nouhi Z, Chughtai N, Hartley S, Cocolakis E, Lebrun JJ (2006). Defining the role of prolactin as an invasion suppressor hormone in breast cancer cells.. Cancer Res.

[91] Barash I (2006). Stat5 in the mammary gland: controlling normal development and cancer.. J Cell Physiol.

[92] Gutzman JH, Rugowski DE, Nikolai SE, Schuler LA (2007). Stat5 activation inhibits prolactin-induced AP-1 activity: distinct prolactin-initiated signals in tumorigenesis dependent on cell context.. Oncogene.

[93] Cotarla I, Ren S, Zhang Y, Gehan E, Singh B (2004). Stat5a is tyrosine phosphorylated and nuclear localized in a high proportion of human breast cancers.. Int J Cancer.

[94] Carsol JL, Gingras S, Simard J (2002). Synergistic action of prolactin (PRL) and androgen on PRL-inducible protein gene expression in human breast cancer cells: a unique model for functional cooperation between signal transducer and activator of transcription-5 and androgen receptor.. Mol Endocrinol.

[95] Fritzsche FR, Thomas A, Winzer KJ, Beyer B, Dankof A (2007). Co-expression and prognostic value of gross cystic disease fluid protein 15 and mammaglobin in primary breast cancer.. Histol Histopathol.

[96] Vandewalle B, Collyn d'Hooghe M, Savary JB, Vilain MO, Peyrat JP (1987). Establishment and characterization of a new cell line (VHB-1) derived from a primary breast carcinoma.. J Cancer Res Clin Oncol.

[97] Imbeaud S, Graudens E, Boulanger V, Barlet X, Zaborski P (2005). Towards standardization of RNA quality assessment using user-independent classifiers of microcapillary electrophoresis traces.. Nucleic Acids Res.

[98] Schroeder A, Mueller O, Stocker S, Salowsky R, Leiber M (2006). The RIN: an RNA integrity number for assigning integrity values to RNA measurements.. BMC Mol Biol.

[99] Maniatis T, Fritsch EF, Sambrook J (1982). Molecular cloning: a laboratory Manual.

[100] Autiero M, Bouchier C, Basmaciogullari S, Zaborski P, El Marhomy S (1997). Isolation from a human seminal vesicle library of the cDNA for gp17, a CD4 binding factor.. Immunogenetics.

[101] Alonso S, Minty A, Bourlet Y, Buckingham M (1986). Comparison of three actin-coding sequences in the mouse; evolutionary relationships between the actin genes of warm-blooded vertebrates.. J Mol Evol.

[102] Graudens E, Boulanger V, Mollard C, Mariage-Samson R, Barlet X (2006). Deciphering cellular states of innate tumor drug responses.. Genome Biol.

[103] ([http://fre2571.vjf.cnrs.fr]) The Genexpress - Array s/IMAGE web site

[104] ([http://image.llnl.gov/]) The I.M.A.G.E. Consortium resources

[105] Brazma A, Hingamp P, Quackenbush J, Sherlock G, Spellman P (2001). Minimum information about a microarray experiment (MIAME)-toward standards for microarray data.. Nat Genet.

[106] ([http://www.ncbi.nlm.nih.gov/geo/]) Gene Expression Omnibus GEO

[107] Hwang D, Schmitt WA, Stephanopoulos G (2002). Determination of minimum sample size and discriminatory expression patterns in microarray data.. Bioinformatics.

[108] (http://www.psycho.uni-duesseldorf.de/abteilungen/aap/gpower3/) GPower3 program

[109] Faul F, Erdfelder E, Lang AG, Buchner A (2007). G*Power 3: a flexible statistical power analysis program for the social, behavioral, and biomedical sciences.. Behav Res Methods.

[110] Eisen MB, Spellman PT, Brown PO, Botstein D (1998). Cluster analysis and display of genome-wide expression patterns.. Proc Natl Acad Sci U S A.

[111] Rhodes DR, Miller JC, Haab BB, Furge KA (2002). CIT: identification of differentially expressed clusters of genes from microarray data.. Bioinformatics.

[112] Benjamini Y, Hochberg Y (1995). Controlling the false discovery rate: a practical and powerful approach to multiple testing. .. J Roy Statist Soc Ser.

[113] Tusher VG, Tibshirani R, Chu G (2001). Significance analysis of microarrays applied to the ionizing radiation response.. Proc Natl Acad Sci U S A.

[114] ([http://www.appliedbiosystems.com]) Applied Biosystems

[115] ([https://www.ingenuity.com]) Ingenuity® Systems

[116] ([http://www.genomatix.de/products/GEMSLauncher/index.html]) Genomatix GEMS Launcher

